# Survey of Tickborne Infections in Denmark

**DOI:** 10.3201/eid1107.041265

**Published:** 2005-07

**Authors:** Sigurdur Skarphédinsson, Per M. Jensen, Kåre Kristiansen

**Affiliations:** * University of Southern Denmark, Odense, Denmark;; † The Royal Veterinary and Agricultural University, Fredriksberg, Denmark;; ‡ Medical Public Health Office, Rønne, Denmark

**Keywords:** Borrelia, Anaplasma phagocytophilum, Bartonella, Rickettsia, Tickborne encephalitis viruses, climate, roe deer, Ixodes ricinus, serology, sentinel surveillance

## Abstract

We conducted a study of the distribution and prevalence of tickborne infections in Denmark by using roe deer as sentinels. Blood samples from 237 roe deer were collected during the 2002–2003 hunting season. Overall, 36.6% of deer were *Borrelia* seropositive, while 95.6% were *Anaplasma phagocytophilum* positive; all animals were negative for *Bartonella quintana* and *B. henselae* by indirect immunofluorescence assay. When a hemagglutination-inhibition test was used, 8.7% of deer were found positive for tickborne encephalitis (TBE)-complex virus. A total of 42.6% were found positive by polymerase chain reaction (PCR) for *A. phagocytophilum* with significant seasonal variation. All were PCR negative for *Rickettsia helvetica*. PCR and sequencing also showed a novel bacterium in roe deer previously only found in ticks. The study showed that the emerging pathogen *A. phagocytophilum* is widely distributed and that a marked shift has occurred in the distribution of TBE-complex virus in Denmark. This finding supports studies that predict alterations in distribution due to climatic changes.

A change in the distribution and frequency of vectorborne infections may be among the first signs of the effect of global climatic change on human health (1). Tickborne infections are the most frequent human vectorborne infections in Europe; the incidence of many of these diseases has been on the rise, and new infections have emerged. In Denmark, borreliosis is known to be endemic and widespread, while tickborne encephalitis (TBE) has been found only on the island of Bornholm. In recent years, human serosurveys have indicated that granulocytic ehrlichiosis caused by *Anaplasma phagocytophilum* is also found in Denmark (2); however, the distribution is unclear. Studies on *Ixodes ricinus* ticks have revealed the existence of other potential pathogens, among them *Rickettsia helvetica* and *Bartonella* spp. (3, S. Skarphédinsson et al., unpub. data). At the same time, increase in the incidence of TBE has been noted in neighboring countries like Germany, Poland, Lithuania, and Sweden (4). Changes in the distribution of TBE in Europe have been suggested to be related to climatic changes, and new foci have been predicted, some within Denmark (5). However, the role of climatic changes is unclear, and increased surveillance is needed to elucidate this in further detail.

*I. ricinus* is the main vector of tickborne infections in Europe and the dominant tick in Denmark (>90%). Roe deer (*Capreolus capreolus*), an important host for *I. ricinus* ticks, have been used as sentinel animals to monitor tickborne infections in several studies, 2 of which have been performed in Denmark. In 1963, Freundt published a survey of TBE (6), and in 1994 Webster and Frandsen evaluated the seroprevalence of *Borrelia burgdorferi* in deer (7). In light of increasing tick density observed, as well as the finding of new pathogens in Denmark, reassessment is indicated (8). The aim of this study was to assess the seroprevalence and geographic distribution of TBE-complex virus, *Borrelia burgdorferi*, *A. phagocytophilum*, *Bartonella quintana*, and *Bartonella henselae* by using roe deer as natural sentinels; at the same time, we evaluated prevalence of infection with *A. phagocytophilum* and *R. helvetica* by using polymerase chain reaction (PCR).

## Materials and Methods

### Sample Collection and Serologic Testing

State forest rangers from the 25 Danish state forest districts were invited to participate during the regular hunting season of 2002 (May 15–July 15, 2002 [summer], and October 1, 2002–January 15, 2003 [fall]). They were asked to obtain blood samples from roe deer by cardiac puncture or from the thoracic cavity when dressing freshly killed animals in the field. For each animal, sex, age, and degree of tick infestation was also noted. Blood collection kits were distributed to all state forest districts by mail, and blood samples were sent by mail to the laboratory. Because of limited amounts of material received from some districts, not all samples were available for all serologic tests.

*B. burgdorferi* serologic tests were performed by using indirect immunofluorescence assay (IFA). *B. burgdorferi* strain DK 6 was used as an antigen, and the conjugate was fluorescein isothiocyanate (FITC)-labeled rabbit anti-deer immunoglobulin (Ig) G (Kierkegaard & Perry Laboratories Inc, Guildford, UK). The cutoff point was 1:64.

*B. henselae* and *B. quintana* serologic tests were performed by using IFA. Slides coated with Vero cells infected with *B. henselae* and *B. quintana* (Focus Technologies, Cypress, CA, USA) were used as antigen, and the conjugate was FITC-labeled rabbit anti-deer IgG (Kierkegaard & Perry Laboratories Inc). The cutoff point was 1:64.

*A. phagocytophilum* serologic testing was performed by using IFA. Slides with HL-60 cells infected with a human isolate of *A. phagocytophilum* (Focus Technologies) were used as antigen, and FITC-labeled rabbit anti-deer IgG was used as conjugate. The cutoff point was 1:128. Slides were considered borderline positive when a definite-but-dim fluorescence equal to that observed for the positive control at its reference endpoint titer was found. Moderate-to-intense fluorescence of the morula was graded positive.

TBE-complex virus serologic testing was performed at the Institute of Virology, University of Vienna, Austria. Samples were first tested by using hemagglutination inhibition (HI) test. Positive samples were verified by using neutralization test.

### DNA extraction, PCR, and Sequencing

Total DNA was extracted from blood samples by using a QIAamp Blood kit (Qiagen, Albertslund, Denmark), according to manufacturer's instructions. Amplifications were performed in a Perkin Elmer GeneAmp PCR system 9600 (Perkin-Elmer Corp, Norwalk, CT, USA), and real-time PCR was performed in Bio-Rad iCycler iQ Quantitative thermal cycler (Bio-Rad, Herlev, Denmark). DNA amplification was done in a 25-μL reaction volume by using ReddyMix PCR Master Mix (ABgene, Epsom, United Kingdom), with 5 μL of sample DNA in each reaction. Cycling conditions included initial 3 min of denaturation at 96°C, followed by 39 cycles, each consisting of 15 s denaturation at 96°C, 15 s annealing at 58°C, and 15 s extension at 72°C. These 39 cycles were followed by an extension period of 3 min at 72°C. Real-time PCR was performed by using HotStartTaq Master Mix kit (Qiagen). Reaction volume was 25 μL with 5 μL sample DNA. Cycling conditions for *Anaplasma* real-time PCR included an initial activation of Taq polymerase at 95°C for 10 min, followed by 40 cycles, each consisting of 15 s denaturation at 95°C followed by 1 min annealing-extension at 60°C. *R. helvetica* real-time PCR included an initial activation of Taq polymerase at 95°C for 10 min followed by 45 cycles of 30 s denaturation at 95°C followed by 45 s annealing-extension at 52°C. Specimen processing, PCR setup, and amplification and detection procedures were all conducted in separate areas to minimize the potential for cross-contamination.

*Anaplasma* infection was detected with primers that specifically target the 16S rRNA gene of the *A. phagocytophilia* genogroup (9): forward primer 5´-GGTACCYACAGAAGAAGTCC and reverse primer 5´-TAGCACTCATCGTTTACAGC. PCR products were detected on 3% agarose gels stained with ethidium bromide. If samples were found positive, a second real-time PCR was performed. Primers specific for the *A. phagocytophilum msp*2 gene were used (10): ApMSP2f (5´-ATGGAAGGTAGTGTTGGTTATGGTATT), ApMSP2r (5´-TTGGTCTTGAAGCGCTCGTA), and a TaqMan probe ApMSP2p-HEX (5´-TGGTGCCAGGGTTGAGCTTGAGATTG). Primers were labeled at the 5´ and 3´ ends with hexachloro-6-carboxy-fluorescein (HEX) and Black Hole Quencher (BHQ), respectively.

*R. helvetica* infection was detected with primers that specifically target the 23S rRNA gene of *R. helvetica*: Rhf (5´-ATAGGGAGGAATTTGAAGGA) and Rhr (5´-GGTAATTTGTACGTCGATCC) and a TaqMan probe Rhpr-TR (5´-CGGAACACAGAACCGTAGCG). Primers were labeled at the 5´ and 3´ ends with Texas Red and BHQ, respectively. For quality control, negative and positive controls were included each time a PCR was performed.

PCR products used for DNA sequencing were purified by using GFX PCR DNA and Gel Band purification kit (Amersham Pharmacia Biotech, Piscataway, NJ, USA). For DNA sequencing reaction, ABI Prism Big Dye Terminator v3.0 kit was used (Perkin Elmer, Applied Biosystems Division). Removal of unincorporated dye terminators was performed by using DyeEx kit (Qiagen) Samples were run on ABI 373A sequencer (Perkin Elmer, Applied Biosystems Division). Sequences were compared with public domain database by using the Blast software. Sequences obtained are available in GenBank under accession nos. AY776165, AY776166, and AY776167.

### Statistical Analysis

Data were analyzed by using STATA 8.2 (StataCorp LP, College Station, TX, USA). For analysis of seroepidemiologic results, Fisher exact test and the Mantel-Haenszel method were used. Values of p<0.05 were considered significant.

## Results

**Figure 1:**
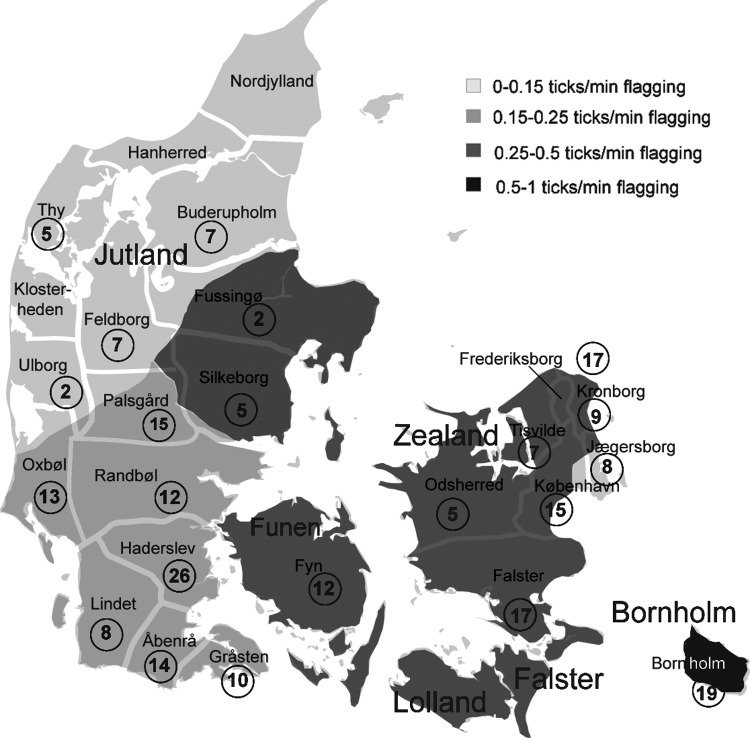
Geographic distribution of roe deer samples collected. The map shows the location of the 25 state forest districts in Denmark. Numbers in circles indicate number of samples collected in each district. Three districts, Klosterheden, Hanherred, and Nordjylland, did not submit samples. Also shown is the approximate density of *Ixodes ricinus* ticks in Denmark, redrawn from (11) as shaded areas. Flagging is the technique of collecting ticks by moving a piece of fabric mounted on a stick through the vegetation for a given period of time.

A total of 237 blood samples from roe deer were collected from 22 of 25 state forest districts (Figure 1). Blood samples from 112 animals were collected in the summer hunting season and from 125 animals in the fall hunting season. The mean age of roe deer was 2.9 years (median 2.5, range 0.5–10 years, Table 1); 60% were bucks, 36% were does, 4% were not defined. This skewed distribution is due to the fact that hunters are only allowed to hunt bucks during the first hunting period in the summer. Only 7% of the roe deer were heavily infested with ticks (defined as >100 engorged ticks/deer); 63% of these animals came from Zealand (p = 0.026).

**Table 1:** Prevalence of Borrelia, Anaplasma phagocytophilum, and tickborne encephalitis (TBE)-complex virus by age group*

Age group	n	*Borrelia* positive (n = 227) (%)	*Anaplasma* positive (n = 227) (%)	*A. phagocytophilum* PCR positive (n = 237) (%)	TBE-complex virus positive (n = 229) (%)
Fawns (≤11 mo)	19	4/17 (23.5)	17/17 (100)	8 (42.1)	0
Yearlings (12–23 mo)	37	13/33 (39.4)	32/35 (91.4)	14 (37.8)	0
Young adults (24–35 mo)	56	13/52 (25.0)	49/51 (96.1)	26 (46.4)	7 (12.5)
Adults (≥36 mo)	100	42 (42.0)	94/99 (94.9)	47 (47.0)	9 (9.0)
Age unknown	25	11 (44.0)	25 (100)	6 (24.0)	4 (16.0)

*Because material received from some state forest districts was limited, not all samples were available for all serologic tests. Where not equal to n, denominator gives actual number of samples tested. PCR, polymerase chain reaction.

### *B. burgdorferi* and *Bartonella* spp.

Seroprevalence was assessed on 227 samples. Of these, 83 (36.6%) had positive results on *Borrelia* IFA, but of these 23 (10%) were borderline positive. Significant regional difference was found in *Borrelia* seropositivity when the mainland of Jutland was compared to the islands (Funen, Zealand, Lolland Falster, Bornholm; Figure 1), 27.1% versus 46.7% (p = 0.003). *Borrelia*-positive roe deer were found in 19 of 22 forest districts evaluated. Three districts in the northern part of Jutland were negative (Fussingø, Thy, and Ulborg; Figure 1), but only 9 blood samples came from these 3 districts. No significant differences in *Borrelia* antibody prevalence were found for sex, age, and season.

*B. henselae* and *B. quintana* seroprevalence was assessed on 227 samples. All samples were seronegative (95% confidence interval [CI] 0%–1.6%)

### A. phagocytophilum

Seroprevalence was assessed on 227 samples. Of these, 217 (95.6%) were positive and 19 were borderline positive (8%). No significant difference in seroprevalence was seen for age, sex, season, or region. Among 237 samples tested by PCR for *A. phagocytophilum*, 101 (42.6%) were positive in both 16S rRNA and *msp*2 PCR analysis. Only samples positive for both genes (16S rRNA and *msp*2) were considered *A. phagocytophilum* positive. Four animals were PCR positive but had negative *Anaplasma* serologic results.

**Figure 2:**
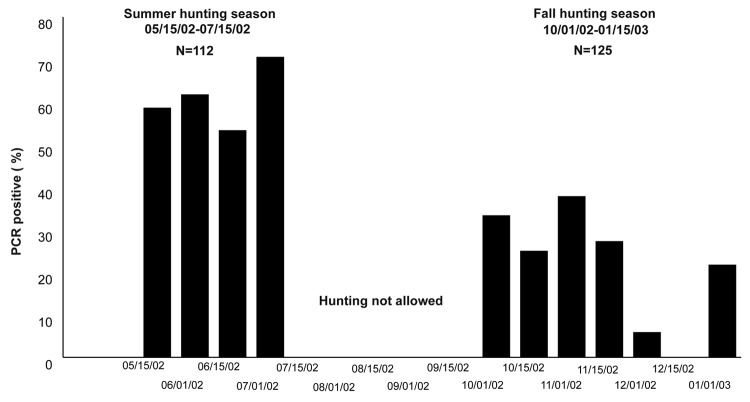
Seasonal variation in samples that were positive on polymerase chain reaction (PCR) for *Anaplasma phagocytophilum*.

Marked seasonal difference was found with 70 (62.5%) positive roe deer during the summer hunting season and 31 (24.8%) positive animals during the fall hunting season (p<0.0001) (Figure 2). Fewer animals were PCR positive in Jutland (39.6%) than on the islands (47.6%), and when adjusted for seasonal difference in sample collection, the difference was significant (p<0.05). *A. phagocytophilum* PCR-positive samples came from all 22 state forest districts (Table 2). Twenty-one samples from Jutland, Funen, Zealand, Falster, and Bornholm that were PCR positive for *A. phagocytophilum* were sequenced (GenBank accession no. AY776165) and revealed 100% homologies with known *A. phagocytophilum* sequences. Sequence variation was only encountered in 1 sample from Oxbøl in Jutland (no. AY776166). No significant difference in sex or age for PCR-positive samples was found. Ten samples were found positive for *Anaplasma* genus on the primary PCR but negative on the more specific secondary PCR. Five of these samples were sequenced (no. AY776167); none was positive for *A. phagocytophilum*, but all had high homology (99%) with a sequence amplified from *I. ricinus* ticks collected from humans in Italy and previously deposited in GenBank as Rickettsiales bacterium it86 (no. AF525482.1).

**Table 2:** Regional distribution of Borrelia-, Anaplasma phagocytophilum–, and tickborne encephalitis (TBE)-complex virus–positive roe deer*

Region + state forest district	n	% *Borrelia* positive†	% *A. phagocytophilum* positive*	% *A. phagocytophilum* PCR positive	% TBE-complex virus HI-test positive
Jutland					
Åbenrå	14	21.4	100	21.4	14.3
Buderupholm	7	71.4	100	30.0	0
Feldborg	7	25.0 (n = 4)	80.0 (n = 5)	14.3	0
Fussingø	2	0	50.0	50.0	0
Gråsten	10	50.0	88.8 (n = 9)	50.0	10
Haderslev	26	16.0 (n = 25)	91.7 (n = 24)	61.5	16.0
Lindet	8	25.0	100	50.0	12.5
Oxbøl	13	46.2	100	53.8	0
Palsgård	15	9.1 (n = 11)	90.9 (n = 11)	20.0	9.1
Randbøl	12	16.7	100	8.3	8.3
Silkeborg	5	60.0	100	80.0	20.0
Thy	5	0	80.0	20.0	0
Ulborg	2	0	100	50.0	0
Funen					
Fyn	12	41.7	100	50.0	0
Zealand-Lolland-Falster					
Falster	17	46.7 (n = 15)	100 (n = 16)	41.2	6.3
Frederiksborg	17	52.9	88.2	52..9	5.8
Jægersborg	8	50.0	100	25.0	12.5
Kronborg	9	55.6	100	66.6	0
København	15	40.0	100	46.6	0
Odsherred	5	80.0	100	80.0	0
Tisvilde	7	42.9	85.7	14.3	0
Bornholm	19	36.8	100	46.2	31.6
Region unknown	2				

*PCR, polymerase chain reaction; HI, hemagglutination inhibition. †Because the amount of material received from some state forest districts was limited, not all samples were available for *Borrelia* and *Anaplasma* serologic testing. Where not all samples were tested, n is indicated in parentheses.

### *R. helvetica* and TBE-complex Virus

**Figure 3:**
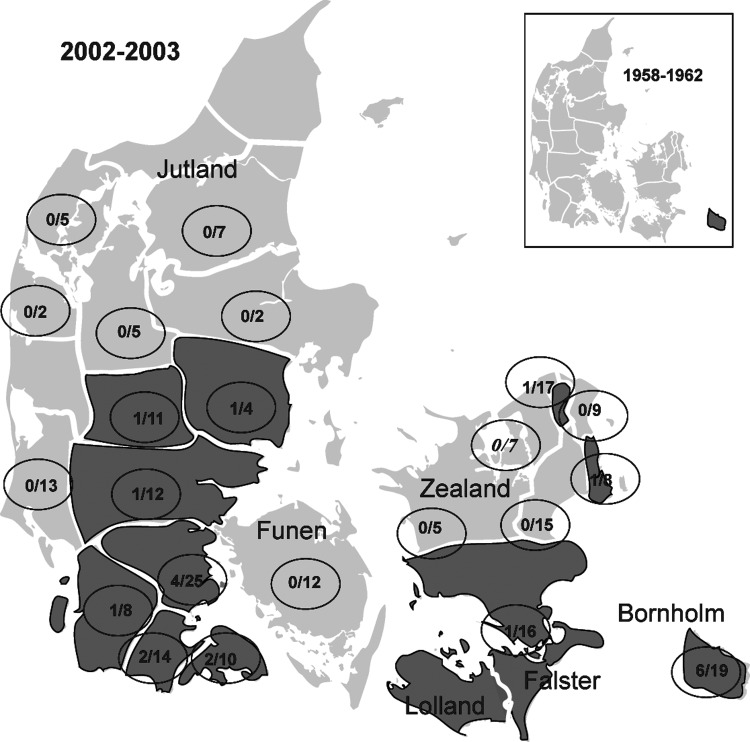
Distribution of tickborne encephalitis (TBE)-complex virus–positive state forest districts (dark shading) in Denmark, 2002–2003 vs. 1958–1962. Numerators indicate number of TBE-complex virus–positive roe deer; denominators indicate number of deer tested.

All 237 samples were tested by PCR for *R. helvetica*, but none were found positive (95% CI 0%–1.6%). Seroprevalence of TBE-complex virus was evaluated on 229 samples. Twenty samples (8.7%) were positive on the HI test. The verifying neutralization test was positive for 14 (6.1%) samples, but 4 could not be evaluated because of cell monolayer destruction, and 2 samples were not available for neutralization testing because of lack of material. Positive samples came from Bornholm (n = 6, 30%), Zealand and Falster (n = 3, 15%), and Jutland (n = 11, 55%) (Figure 3). Significant differences in seroprevalence could not be shown for sex or season, but TBE-complex virus–positive animals were significantly more common in the young adult–adult group than in the fawn–yearling group (p = 0.014).

## Discussion

In the present study, roe deer were used as sentinels. They are widely distributed throughout Denmark and outnumber other large wild animals. They play a central role in the life cycle of *I. ricinus* by feeding large numbers of the tick at all 3 life stages (12). Density of *I. ricinus* is strongly related to the abundance of roe deer (13,14). Roe deer are considered incompetent as reservoirs of *Borrelia* spp (15); however, their central role in the life cycle of the tick and the fact that they can respond immunologically to *Borrelia* infection renders them useful as sentinels for borreliosis (16). The finding of a *Borrelia* seroprevalence of 36.6% is lower than that found in the study by Webster and Frandsen (7) from 1994 (52%), but in the previous study all roe deer samples came from Zealand, and when this regional difference is taken into account the difference is not significant (50% vs. 52%, p = 0.773). The regional differences found correlate well with the known tick density in Denmark (Figure 1). The lack of seropositive roe deer from 3 districts in northern Jutland may, however, be due to the low sample size from these districts.

The role of ticks and roe deer in the transmission of *Bartonella* infections is still uncertain. Schouls et al. found that 60% of ticks collected from infested roe deer carried *Bartonella* spp (17). *B. henselae* has also recently been found in ticks infesting humans in Italy (18), and *B. quintana* has been recovered from ticks in California (19). In 2001, McGill et al. found that 31% of elite orienteers (participants in the competitive sport of finding the fastest route between defined checkpoints with only a compass and map), a high-risk group for tick bites, were seropositive to *Bartonella elizabethae* (20). The recent finding of patients in the United States with *Borrelia burgdorferi* and *B. henselae* co-infection also supports the idea that ticks play a role as a vector for *Bartonella* spp (21). Our finding of no *B. henselae*– or *B. quintana*–seropositive roe deer does not support this idea. Whether roe deer are seropositive to other *Bartonella* spp., like *B. elizabethae*, remains to be evaluated, as well as the human pathogenic potential of *B. elizabethae*.

Roe deer are thought to be competent reservoirs of *A. phagocytophilum* (22), and a high seroprevalence has been found in previous studies in Europe. We found a seroprevalence of 95.6%, which is similar to findings from Norway (96%) and Slovenia (94%) (23,24). Larger variation has been found in the number of PCR-positive roe deer, from 12.5% in Czech Republic to 85.6% in Slovenia (24,25). Although differences in PCR protocols may to some extent explain this difference, the seasonal variation in the number of PCR-positive roe deer, as shown in this study, may also play a role (Figure 2). As the probability of roe deer being rickettsemic changes through the season, serology represents a better surveillance tool than PCR. The high proportion of roe deer that are bacteremic throughout the tick season and the importance of roe deer in the life cycle of *I. ricinus* may indicate that roe deer are the main reservoir of *A. phagocytophilum* in Europe.

Although regional variation was found in the number of PCR-positive animals, *A. phagocytophilum* is widespread in Denmark and seems correlated to tick and roe deer density. Limited variation was found among the 16S RNA sequences analyzed. Reliable differentiation of possible *A. phagocytophilum* strains in Denmark would be better accomplished by sequencing genes with higher variation than the conserved sequence of 16S RNA; among better candidates are the Ank-gene and the groESL operon. Further characterization of European strains of *A. phagocytophilum* is needed; the finding of a high *A. phagocytophilum* prevalence in roe deer compared to the low number of human anaplasmosis cases reported in Europe may indicate the existence of strains less virulent or nonpathogenic to humans (26). The finding of sequences in roe deer blood that are related to sequences previously amplified only from ticks in Italy (It86-Belluno) is of interest and should be studied further (27). Whether this organism is pathogenic to roe deer or can cause human infection remains to be elucidated. The accuracy of diagnostic assays used is critical to any pathogen surveillance, and the potential for serologic cross-reaction is an important consideration. Recently *R. helvetica* has been found in ticks from Bornholm and Jutland, and a seroprevalence of 12.5% was described in high-risk groups in northern Jutland (3, S. Skarphédinsson et al., unpub. data). Even though it is not phylogenetically close to *A. phagocytophilum*, and even though serologic cross-reactivity has not been reported to date, it is the only other *Rickettsia* species reported in Denmark. We therefore looked for *R. helvetica* in roe deer, but found no PCR-positive deer. The explanation for this finding may be that roe deer are not competent as reservoirs or that the bacteremic phase is very short. Another possibility is that *R. helvetica* has a very focal distribution or is even disappearing from Denmark, as the seroprevalence study of Nielsen et al. showed a gradual decrease in seroprevalence from 29% in 1997 to 0% in 2000 (3).

Tickborne encephalitis was the first tickborne infection to be recognized in Denmark. During the years 1958–1962, E.A. Freundt did a survey for TBE-complex virus using human and animal sera from all parts of Denmark. He found, using both HI, neutralization, and complement fixation tests, that TBE-complex virus was present only on the island of Bornholm. He found an overall seroprevalence of 8.6% in Danish roe deer (the local seroprevalence on Bornholm was 83%) (6). Since then a very limited surveillance of TBE has since been carried out in Denmark. Recent increases in TBE cases in neighboring Sweden have been suggested to be related to climatic changes (28), as milder climate has been followed by a northern shift in the distribution limit of *I. ricinus* as well as a general increase in tick density (29). However, the variable patterns of changing TBE case numbers in Europe indicate that changing climate is not the sole causal factor. Changes in the densities of hosts for ticks and sociopolitical circumstances may play a role as well (30). TBE has also been suggested by the World Health Organization–European Centre for Environment and Health working group on the early implication of climatic change to be a priority infection for surveillance during climatic change (31) because of the fragile and temperature-dependent natural cycle of TBE virus. Using geographic information systems and remote sensing, Randolph et al. have predicted the present as well as future distribution of TBE in northern Europe with changing climate (32). These predictions seem to correlate well with the findings in our study of a change in the distribution of TBE-complex virus in Denmark. A strict correlation between TBE-complex virus–positive areas and tick and roe deer density was, on the other hand, not found.

However, recent studies on ticks in Bornholm have shown that not only the Western European subtype of TBE-complex virus is to be found. Louping ill virus, another flavivirus belonging to the TBE antigenic complex, is now also found in Bornholm (33). Further studies are needed to clarify the possible role of serologic cross-reactivity between these 2 closely related viruses. Although we found the same overall TBE seroprevalence now as in 1962, the local seroprevalence in Bornholm is significantly lower than before (31.6% vs. 83%, p = 0.001). Whether the emergence of Louping ill virus plays a part in this decrease is of interest. The fact that only roe deer 24 months of age or older were TBE-complex virus–seropositive may indicate that although TBE-complex virus has emerged in new areas in Denmark, the infection is still rare and focal in distribution.

Using roe deer as sentinels, we have shown that *A. phagocytophilum* is now widely distributed in Denmark and that roe deer may be the main reservoir. Also, while *Borrelia* prevalence has remained stable, the distribution of TBE-complex virus has changed, which supports the predicted effect of climatic change on vectorborne infections in northern Europe. In a shifting climate, continued long-term monitoring of tickborne infections is of importance. Healthcare providers should also be aware of the dynamic changes in distribution and prevalence of these infections when treating a patient with compatible illness, specifically after exposure to ticks.
